# The potential consequences for cancer care and cancer research of Brexit

**DOI:** 10.3332/ecancer.2017.ed63

**Published:** 2017-02-24

**Authors:** Peter Selby, Mark Lawler, Richard Baird, Ian Banks, Patrick Johnston, Paul Nurse

**Affiliations:** 1 University of Leeds, Section of Oncology and Clinical Research, Cancer Research Building, St James’s University Hospital, Beckett Street, LEEDS LS9 7TF, UK; 2 Association of Cancer Physicians (UK); 3 European Cancer Concord; 4 Translational Cancer Genomics, Centre for Cancer Research and Cell Biology, Queens University Belfast, University Road, Belfast, BT7 1NN, UK; 5 Research and Innovation, European Cancer Concord; 6 Breast Cancer Therapeutics, Cambridge Breast Cancer Research Unit, UK; 7 Patient Advocacy, European Cancer Concord; 8 Queens University Belfast, University Road, Belfast, BT7 1NN, UK; 9 Francis Crick Institute, 1 Midland Rd, Kings Cross, London, NW1 1AT, UK; * *joint first authors*

**Keywords:** cancer research, cancer care, Brexit, European Union

## Abstract

Following the UK “Brexit” vote in June 2016, there are many uncertainties and risks for cancer research and cancer care in the UK. These are summarised and the importance of sustained engagement and influence from the cancer community on UK governments is emphasised.

On 23 June 2016, the United Kingdom (UK) voted by a small margin to leave the European Union (EU). The consequences of that vote are as yet far from clear. There have been considerable political and economic upheavals but, in fact, the process of leaving the EU has yet to formally begin. The government expects to initiate the process in the Spring of 2017 and for it to continue for at least two years of negotiations and the Prime Minister has stated that it is her intention that we leave the EU internal market. In the absence of a clear picture of the terms upon which the UK will exit the EU, it is difficult to update the likely impact on cancer research and cancer patients with any confidence. Prior to the vote, we argued that the EU provided important elements of support for the excellence of UK cancer research including funding, movement of cancer researchers of all seniorities between the UK and the EU and an important voice in research healthcare strategy in Europe. We expressed lack of confidence that if these were lost as a result of leaving the EU they may not be replaced by UK government [1]. The jury remains firmly “out” as to the consequences of an EU exit for cancer research and cancer care, but there are widely held concerns among the cancer community.

Some reassurance has been offered. It has been agreed that current EU grant funding held by UK individuals and institutions will be honoured. In addition, the UK government is talking about increasing the total spend available for research, within which cancer might figure significantly.

The complexity of the separation of the UK from the EU for cancer research is considerable. It includes the potential for change in existing Regulations, Directives and Laws that can influence cancer research and are derived from EU legislation adopted into UK law. Nothing has changed at the moment and nothing will change until the UK’s intention to withdraw from the EU is stated officially and there is a two year period within which the terms will be settled by negotiation. It is possible that this period of negotiation will be extended by the parties involved. Clearly, at the time of any exit, there will have to be in place a legal framework to replace that which has been provided through the EU. EU Regulations which are directly applied in Member States, would effectively “disappear” at the time of an exit from the EU. If the UK government wishes to continue to abide by these regulations, then they would have to incorporate them into UK law. The UK’s responses to EU Directives may well have been already been passed as national legislation and would continue following an exit.

Examples of direct relevance to clinical cancer research include the EU Clinical Trials Directive, first published in 2001. The UK put in place formal clinical trials regulations in 2004 and amended them in 2006, 2008 and 2009. Following the EU Clinical Trials Directive, there was a fall in the number of clinical trials, widely blamed on increased costs and delays in launching trials. The EU is in the process of developing a new regulation which is expected in 2018. After Brexit, the UK will have to establish its own clinical trials regulatory environment which will require careful design to achieve the minimum necessary bureaucracy and compatibility with other European countries for multi-national trials. A similar situation applies to the EU Good Manufacturing Practice Directive launched in 2003. The UK created regulations bringing this into place and amended them in 2003, 2007 and 2008. This Directive covered medical devices and diagnostic tests. Brexit would mean that the EU Directive no longer applied in the UK, which is of concern to the manufacturers of devices and tests unless the EU and the UK share regulatory frameworks in future to allow ready import and export of devices/tests. The EU issued a Good Clinical Practice (GCP) Directive in 2005 which entered UK Regulation in 2006. Although the EU Directive stimulated the UK legislation, the principles of GCP are widely accepted internationally and are likely to be incorporated into UK law.

The European Medicines Agency (EMA) has its headquarters in the UK and oversees the routes to obtain market authorisation throughout the EU through a variety of procedures. These authorisations may be centralised or operated in one or more countries. Brexit would mean that the UK does not have access to the centralised authorisation procedures although other countries (eg Switzerland) have negotiated close relationships. It is also possible that the EMA would not feel it was appropriate to have its headquarters in a country not fully part of the EU. If the EMA were to move to another jurisdiction, then the Medicines and Healthcare products Regulatory Agency (MHRA) would have to step up to take over certain regulatory activities and conduct cooperations with other global regulators.

The situation now is fluid in relation to Data Protection. The EU has proposed a new General Data Protection Regulation (Regulation (EU) 2016/679) which will come into force in 2018. It is possible that this would be applicable in the UK for a short period. There would be considerable uncertainty about the movement of personal research data between the EU and the UK. Data protection regulations would influence UK companies wishing to trade in services to individuals within the EU. All of this is likely to be the subject of complex negotiations. The Human Tissue Act 2004 and the Mental Capacity Act 2005 which affect clinical practice and research in cancer significantly are not EU derived and would be unaffected by Brexit.

Research funding streams from the EU are open to non-EU countries under a variety of negotiations and conditions. It is possible that the UK would be able to remain part of these and even conceivable that it would remain possible for the UK to “punch above its weight” in terms of capturing the EU funding. However, it is equally possible that there would be a significant deficit in the amount of funding that UK researchers could attract from the EU. Additionally, the uncertainty about research funding may discourage senior researchers from relocating to the UK, particularly if they felt that prestigious funding like European Research Council grants and Horizon 2020 were no longer available to them. Anecdotally, a number of UK institutions have already indicated that pre-Brexit recruits have turned down positions due to uncertainties related to continued EU funding.

## Conclusion

This overview highlights the potential complexities in relation to the laws and regulations that are relevant to cancer research, many of which are derived from EU Directives/Regulations. It is important that the UK’s cancer researchers and cancer care providers watch the “Brexit space” very closely. We need to take every opportunity to remind the UK government that cancer patients and cancer research could be significantly disadvantaged, unless great care is deployed in the negotiations of the legal and economic frameworks which will govern the UK’s relationship with the EU beyond Brexit. When the Brexit negotiations are complete, the UK government may be prepared to step up to efficiently fill the gaps in law, regulation, resource allocation and strategic alliances that will probably ensue. If they do not do so, cancer research and cancer patients will suffer.
